# The magnitude of chronic constipation and associated factors among type 2 diabetic patients in Harar, Eastern Ethiopia

**DOI:** 10.1186/s40842-024-00188-3

**Published:** 2024-10-17

**Authors:** Wubshet Nebiyu Mogess, Tefera Belsty Mihretie, Mezgebu Legesse Habte, Teka Obsa Feyisa, Bilisuma Girma Areda, Ebsa Tofik Ahmed, Getahun Chala Diribsa, Mastewal Zeleke, Natan Muluberhan Alemseged, Eyobel Amentie, Tegenu Balcha Wodajo, Tewekel Reshid Borushe

**Affiliations:** 1 Department of Medical Anatomy, School of Medicine, College of Health and Medical Sciences, Haramaya University, Harar, Ethiopia; 2 Department of Medical Biochemistry, School of Medicine, College of Health and Medical Sciences, Haramaya University, Harar, Ethiopia; 3 Department of Medical Physiology, School of Medicine, College of Health and Medical Sciences, Haramaya University, Harar, Ethiopia; 4 Department of Emergency Medicine and Critical Care, School of Medicine, College of Health and Medical Sciences, Haramaya University, Harar, Ethiopia; 5 Department of Surgery, School of Medicine, College of Health and Medical Sciences, Haramaya University, Harar, Ethiopia; 6 Department of Nursing, School of Medicine, College of Health and Medical Sciences, Haramaya University, Harar, Ethiopia

**Keywords:** Chronic constipation, Rome III criteria, Type2 diabetes

## Abstract

**Background:**

Constipation, which affects 16% of adults worldwide, is a chronic health problem characterized by unsatisfactory frequency of bowel movements, causing pain, bloating or incomplete bowel movements. The study aims to assess the magnitude of chronic constipation and associated factors among T_2_DM patients attending the endocrinology outpatient clinic at Hiwot Fana Comprehensive Specialized University Hospital from January 1 to May 30, 2023.

**Methods:**

Hospital-based cross-sectional study design was carried out to assess the magnitude and associated factors of chronic constipation among T_2_DM patients at Hiwot Fana Comprehensive Specialized University Hospital. Using a single population formula 300 T_2_DM patients were enrolled in this study. The data was analyzed by using the Epi-Data 4.6 and SPSS version 25. Descriptive, bivariate, multivariate, and logistic regression were used. *P* < 0.05 was used to declare association.

**Results:**

300 T2DM patients participated in this survey. Of these 137 (45.7%) were male and 163 (54.3%) were female and the mean age was 58.57 ± 11.09 SD years, the range from 35 to 85 years. The prevalence of constipation was 73 (24.3%) (95% CI: 0.196–0.296). Education status above high school (AOR: 0.151.95% CI: 0.032–0.718), less than 7 h of sleep per day (AOR: 12.39.95% CI: 2.712–56.69), frequent depression (AOR: 6, 84, 95% CI: 2.639–17.743), parents with constipation (AOR: 6.843.95% CI: 2.639–17.743), daily water intake < 1300 ml (AOR: 4.760.95% CI: 1.146–19.773), TAG levels below 150 mg/dl (AOR: 0.050, 95% CI: 0.015–0.166), HbAlc between 6 and 7% (AOR: 0.013.95% CI: 0.001–0.132) ,HbAlc between 7.1 and 8% (AOR: 0.006, 95% CI: 0.001–0.067), and LDL levels were significantly associated with chronic constipation in T2DM patients.

**Conclusions:**

The prevalence of chronic constipation was considerable in T2DM patients. Education level above high school, less than 7 h of sleep per day, frequent depression, parents with constipation, daily water intake < 1300 ml, TAG and HbAlc play a significant role in the development of chronic constipation in T2DM patients. T2DM patients can reduce the extent of constipation by treating the above problem in a timely and timely manner.

## Background

Constipation is a chronic health problem estimated to affect up to 16% of the world’s adult population [1, 2]. The term constipation refers to an unsatisfactory frequency of bowel movements, which may be caused by hard, painful, or difficult bowel movements, difficulty passing gas, or a feeling of incomplete bowel movements [2, 3]. The frequency and incidence of constipation vary depending on independent variables such as the age and race of study participants as well as variations in diagnostic criteria [4]. Numerous factors can lead to chronic constipation, including lack of physical activity, inadequate fluid intake, medication side effects, previous surgery, the insufficient fiber in the diet, excessive intake of foods that cause constipation, and chronic illness [5].

Type 2 diabetes mellitus (T2DM) is a severe global disease characterized by hyperglycemia caused by gradually losing the ability to produce enough insulin associated with insulin resistance [6]. Type 2 diabetes (T2DM) is a chronic disease that is becoming increasingly common worldwide and represents a major health problem that is expected to increase in the future [7]. Type 2 diabetes (T2DM) accounts for 90% of all cases diagnosed in Africa [8]. The prevalence of diabetes mellitus (DM) in Ethiopia is higher than in other African countries and is occasionally increasing at a worrying rate [9]. The long-term effect of T2DM damages almost every organ in the human body. In diabetics,, diabetic foot, diabetic retinopathy, and diabetic nephropathy are the most common. They are also at risk of tachycardia, orthostatic hypotension, indigestion, nausea, diarrhea and/or constipation, and urinary incontinence [10].

Constipation is one of the problems that T2DM patients suffer from, and research has shown that these patients suffer from constipation more often than the general population [11].

Some studies emphasize the effect of DM on the colon’s function, particularly the lower gastrointestinal tract. DM has the potential to alter colonic motility and visceral sensation by affecting the structure, function, and density of the epithelial cells of the gastrointestinal system [12]. Various studies suggest that the severity of constipation in diabetics i**s** related to the effect of DM on the autonomic nervous system. More than 50% of diabetic outpatients suffer from constipation and up to 20% of them require laxative treatment [13].

Patients with Chronic constipation suffer greatly from stress, which affects patients’ quality of life and places a significant burden on the healthcare system [14, 15]. Constipation and the specific clinical features of patients with diabetes have not been widely investigated in reports [4].

In a single study conducted in Dessie, Ethiopia, 16% of diabetics reported suffering from constipation [16]. The delay in diagnosing constipation is compounded by the fact that its symptoms can resemble those of gastroparesis and can also be confused with the side effects of diabetes medications. However, it is important to detect constipation early in order to effectively treat the symptoms and avoid possible complications. Accurate information about the incidence of chronic constipation in T2DM is critical for adapting diabetes care policies and practices to improve disease control.

Therefore, there are not enough studies on the prevalence of chronic constipation in T2DM patients and its contributing factors in Ethiopia. To address this gap in the study, this study aimed to assess the magnitude of chronic constipation and its associated factors in T_2_DM at Hiwot Fana Comprehensive Specialized University Hospital.

## Methods

### Study area, design, and period

This is a cross-sectional study conducted from January 1 to May 30, 2023 on 300 participants at Hiwot Fana Comprehensive Specialized University Hospital Harar, Eastern Ethiopia. The city of Harar is located 526 km away from the capital city of Ethiopia, Addis Ababa. The hospital is a large teaching hospital under the management of Haramaya University. The hospital has four main departments (Medicine, Surgery, Pediatrics, and Gynecology-Obstetrics) as well as other smaller departments. The hospital provides services for more than 20 million Population in Eastern Ethiopia. And also give teaching services for public and private college students.

### Inclusion and exclusion criteria

This study consisted of all T2DM patients who came to Hiwot Fana Comprehensive Specialized University Hospital for follow-up during the study period. All T2DM patients who received at least one antidiabetic drug for at least three months during the data collection period were included in the study. T2DM patients with known liver disease, pregnant women or breastfeeding, and previous major intra-abdominal surgery were excluded from the study.

### Sample size determination

The sample size of the study was calculated based on the single population formula by assuming that DM prevalence is the highest population proportion of constipation prevalence among adults, which is 32% [17]with a 95% confidence level and 10% non-response rate. Finally, 300 sample sizes were included in this study.

### Data collection

A structured and pretested questionnaire was administered. Sociodemographic data were collected through personal interviews using a structured questionnaire. The English version of the questionnaire was prepared from published articles and expertly translated into the native languages of Amharic and Afan Oromo. Information was collected by two nurses who completed a one-day training program. Data collection procedures were supervised by the principal investigator and each co-investigator. Trained nurses obtained all relevant health, sociodemographic, and anthropometric data as well as a 5-milliliter fasting blood sample from each eligible study participant after receiving a completed consent form from the hospital director and the T2DM patient. The criteria from Room III [18] and the Bristol scale chart [19] were used to assess the frequency of constipation. The blood sample was collected using serum separator test tubes. A fully automated chemistry analyzer was used to measure the lipid profile and fasting blood glucose.

### Data analysis

For analysis, the collected data were entered into Epi-Data 4.6 and exported to SPSS 25.0. Categorical variables were described as counts, percentages, and numerical variables as means ± standard deviations. The chi-square test is used to compare differences in the prevalence of chronic constipation based on independent variables. To find out the statistical significance, logistic regressions were performed with a confidence interval (CI) of 95%. Binary logistic regression analysis was conducted to calculate the crude odds ratio (COR). The variables found to be significant or slightly insignificant according to the COR value (p-value less than or equal to 0.25) were entered into the multiple logistic regression and the adjusted odd ratio of the determinants of chronic constipation occurrence was used. Model fitness was tested using Hosmer and Leeshawn model fitting and variable multicollinearity was checked using the variance inflation factor. Finally, variables were considered statistically significant if their p-value was less than 0.05.

## Results

Of the total 300 participants, 137 (45.7%) were males and 163 (54.3%) were female. The mean age of the participants was 58.57 ± 11.09 years with a range of 35 to 85 years. About 92% of study participants were involved in privately employed (Table 1).The educational profile of the study participants showed that most of participants 112 (37.3%), were unable to read and write, while 43(14.3%) complete Diploma and above (Table 1). In this study, about 288 (96%), 297(99%), and (206) 68.7% of the study participants were non-alcohol drinkers, non-smokers, and non-chat chewers, respectively. About 64% of the study participants engage in regular physical activity at least three times a week. According to this study, about 72.3% of participants had good fiber intake while 27.3% had poor fiber intake. Study participant’s hours of sleep per day showed that 28.3% slept less than 7 h, 56.7% slept 7 to 9 h, and the remaining 15% slept more than 9 h per day. Metformin is the most commonly used hypoglycemic drug (62.7%) followed by insulin (25.3%). More than 50% of T2DM patients in this study used statin (Table 1).

The study revealed that the prevalence of chronic constipation in T_2_DM patients according to Rome III criteria was 73/300 (24.3%). Of these, 11% were male and 13.3% of the patients were female.

**Table 1:** Sociodemographic characteristics and lifestyle of T2DM patients attending Hiwot Fana Comprehensive Specialized University Hospital, Harar, Ethiopia, 2023

Variable	Categories	Constipation		Frequency (%)
		No	Yes	
Sex	Male	104	33	137(45.7%)
	Female	123	40	163(54.3%)
Age	30–39	9	1	10(3.3%)
	40–49	46	10	56(18.7%)
	50–59	65	16	81(27%)
	>=60	107	46	153(51%)
Occupation	Government	17	6	23 (7.7%)
	Private	209	67	1(0.3%)
	NGO	1	0	276(92%)
Educational status	Unable to read and write	75	37	112(37.3%)
	Primary school	78	17	95(31.7%)
	High school	42	8	50(16.7%)
	Diploma and above	32	11	43(14.3%)
Alcohol intake	Drunker	9	3	12(4%)
	Non-drunker	218	70	288(96%)
Cigarette smoking	Smoker	1	0	1(0.3%)
	Nonsmoker	225	72	297(99%)
	Quite smoke	1	1	2(0.7%)
Chat chewing	Chewer	72	22	94(31.3%)
	Non-chewer	155	51	206(68.7%)
Regular physical exercise	No	78	30	108(36%)
	Yes	149	43	192(64%)
Fiber intake status	Poor	59	23	82(27.3%)
	Good	167	50	217(72.3%)
	Excellent	1	0	1(0.3%)
Sleep hour per day	Less than 7 h	33	52	85(28.3%)
	7–9 h	152	18	170(56.7%)
	Greater than 9 h	42	3	45(15.0%)
Type hypoglycemic drug used	Metformin	143	45	188(62.7%)
	Insulin	28	8	36(12%)
	Combined	56	20	76(25.3%)
Statin utilization	Yes	113	44	157(52.3%)
	No	114	29	143(47.7%)
Body mass index	Underweight	5	3	8(2.7%)
	Normal	109	32	141(47%)
	Overweight	90	28	118(39.3%)
	Obese	23	10	33(11%)

### Associated factors affecting constipation among T2DM patients

#### Sociodemographic and other related lifestyle factors

This study revealed that educational status, hours of sleep per day, frequent depression, and daily water intake were statistically significant risk factors for chronic constipation in T_2_DM patients (Table 2).

According to multiple logistic regression results, patients who have completed high school or higher are 0.15 less likely to have constipation than those who cannot read or write. The result showed that patients who sleep less than seven hours per day were 12.39 times more likely to suffer from chronic constipation than patients who sleep more than nine hours per day (OR = 12.39, 95% CI: 2.712–56.69). The results indicated that consuming less than 1300 ml of diluted water can increase constipation 4.760 times more than consuming more than 2700 ml of water per day (OR = 4.760, 95% CI: 1.146–19.773) (Table 2).

### Clinical and lipid profile factors

Patients from parents with constipation, depression, LDL cholesterol, triglyceride, TAG and HbAlc were statistically significant risk factors for chronic constipation in T_2_DM patients.

The likelihood of constipation is 6.843 times higher in frequently depressed T2DM patients than in non-depressed patients. The odds of constipation are increased by 30.102 times from T2DM patients whose parents are affected by constipation (Table 2).

The likelihood of constipation decreases 0.061 times with an LDL level of less than 100 compared to an LDL level of more than 190 mg/dl (Table 2). Constipation is 0.05 times less likely in T2DM patients with TAG less than 150 mg/dl than in patients with 200–499 mg/dL (OR = 0.05, 95% CI: 0.015–0.166, *P* = 0.001). Constipation is 0.013 times less in patients with 6–7% HbAlc than in patients with 8% (Table 2).

**Table 2:** Multivariate binary logistic regression analysis for associated factors affecting constipation among T_2_DM patients attending Hiwot Fana Comprehensive Specialized University Hospital, Harar, Ethiopia, 2023

Variable	Categories	Constipation		Odds ratio (95%CI)				*P*-value
		No	Yes	COR		AOR		
Age	30–39	9	1	0.258(0.032,2.099)		0.355 (0.02–6.255)		0.48
	40–49	46	10	0.506 (0.235–1.088)		1.058(0.338–3.310)		0.92
	50–59	65	16	0.573 (0.300-1.0930		0.543(0.191–1.543)		0.252
	>=60	107	46	1		1		
Educational status	Unable to read and write	42	8	1.437(0.65–3.16)		0.634(0.188–2.145)		0.46
	Primary school	78	17	0.634(0.268–1.503)		0.369(0.099–1.373)		0.13
	High school	32	11	0.554(0.200-1.537)		0.151(0.032–0.718)		0.017*
	Diploma and above	75	37	1		1		
How long sleep per day	Less than 7 h	42	3	22.06(6.32–76.9)		12.39(2.712–56.69)		0.001*
	7–9 h	152	18	1.658(0.46–5.89)		0.901(0.186–4.362)		0.89
	> 9 h	33	52	1		1		
Frequent depression	Yes	205	42	6.87(3.62–13.03)		6.843(2.639–17.743)		0.0001*
	No	22	31	1		1		
Parents with constipation	Yes	10	39	24.89(11.37–54.47)		30.102(10.503–86.277)		0.0001*
	No	217	34	1		1		
Daily water intake	< 1300 ml	41	6	4.698(1.791–12.326)		4.760(1.146–19.773)		0.032*
	1300–2700	138	34	1.684(0.661–4.290)		1.609(0.425–6.087)		0.48
	>=2700	48	33	1		1		
LDL(mg/dl)	< 100	86	4	0.014(0.004–0.05)		0.061(0.012-0.300)		0.001*
	100–129	70	10	0.044(0.016–0.120)		0.144(0.034–0.621)		0.001*
	130–159	62	30	0.150(0.063–0.357)		0.498(0.143–1.735)		0.009*
	>=190	9	29	1		1		
TAG(mg/dl)	< 150	178	9	0.019(0.007–0.049)		0.050(0.015–0.166)		0.001*
	150–199	38	34	0.328(0.143–0.753)		0.518(0.169–1.591)		0.251
	200–499	11	30	1		1		
HDL(mg/dl)	< 40	7	1	4.273(0.510-35.828)		3.801(0.227–52.154)		0.318
	40–59	142	25	1.232(0.145–10.453)		1.485(0.105–20.948)		0.770
	>=60	77	47	1		1		
HbAlc (%)	6–7	82	1	0.013(0.002–0.095)		0.013(0.001–0.132)		0.001*
	7.1-8	70	1	0.015(0.015–0.112)		0.006(0.001–0.067)		0.001*
	> 8	75	73	1		1		
TC	< 200	218	38	0.087(0.021–0.364)		0.508(0.067–3.433)		0.488
	200–239	6	29	2.417	0.468–12.473	3.411(0.417–27.912)		0.253
	>=240	3	6	1		1		

CI: Confidence Interval; HbA1c: Glycated Hemoglobin; HDL: High Density Lipoprotein; LDL: Low Density Lipoprotein; OR: Odds Ratio: *: Statistically Significant, TC: Total Cholesterol, TAG: Triacylglycerol, ml: milliliter, mg/dl: milligram per deciliter

## Discussion

Chronic constipation is the lack of satisfactory bowel movements, it is a common gastric motility disorder worldwide Due to different definitions and diagnostic techniques, there are different reports on the occurrence of chronic constipation in different populations and countries [20]. Studies have shown that non-communicable chronic diseases such as diabetes can affect gastrointestinal health and increase the prevalence of chronic constipation [21]. Our result showed, that the prevalence of chronic constipation in T_2_DM patients according to Rome III criteria was 24.3%, of these, 11% were male and 13.3% were female. This finding is consistent with studies in India 24.7% [22]. However, the prevalence of this study is lower than the finding in Nigeria 27% [23] and Australia 28% [24]. The discrepancy might be due to differences in sample size and study design Our finding revealed that the prevalence of constipation is higher in females than males [21]. These results are supported by a review done by Neto MÁ et al. [8]. In addition, sleeping less than seven hours per day could increase likelihood of constipation by 12.39 times more than those who sleep more than nine hours per day. This result is in line with the study done in the US which showed that constipation is highly common among those who sleep less than seven hours per day [25]. This might be due to short sleeping hours can alter circadian rhythms of gastrointestinal function by increasing abnormal colon motion [26]. Short periods of sleep increase constipation by altering the average levels of the inflammatory markers interleukin-6 and C as confirmed by an experimental study in Iran [27]. Our result indicated that constipation is 6.843 times more likely in depressed T2DM patients than in non-depressed patients. This finding is comparable to a study of American adults that reported that constipation was three times more common in depressed adults [28]. This is also supported by a study conducted among nonacademic staff at the University of Isfahan in Iran [29]. This could be justified because the brain plays an important role in emotional stress and motor function of the upper and lower gastrointestinal system [30]. Our finding indicates that the odds of constipation are 30.302 more likely among parents who have a history of constipation. This finding is consistent with a study conducted in China [31]. This study also showed that the risk of constipation is 4.67 times higher in diabetics who consume less than 1300 ml water per day than those who consume more than 2700 ml water. This result is comparable to other studies in Japan that report lower water intake as a risk factor for constipation [32]. This is also supported by a study conducted in Turkey, which reported that constipation is likely to increase in patients who do not drink enough water [33]. This may be due to low water intake, which makes bowel movements difficult and reduces the amount of stool, leading to constipation [34]. In this study, constipation is less likely for diabetic patients who had less than 100 mg/dl LDL as compared to diabetic patients with LDL greater than 190 mg/dl. It indicates that an increase in serum LDL can increase likelihood of constipation among diabetic patients. This finding is consistent with other comparable studies reporting that an increase in low-density lipoprotein (LDL) may increase the likelihood of constipation [23, 24]. According to our results, diabetic patients with a serum TAG value of less than 50 mg/dl were less likely to suffer from constipation than patients with a TAG value of 200-499 mg/dl. This is supported by a previous study that reported that increasing the amount of TAG may increase the risk of constipation [25]. The serum concentration of HbA1c in T_2_DM patients indicates how well they control their blood glucose. According to this study, higher serum HbA1c among T_2_DM patients is a statistically significant associated factor for chronic constipation in T_2_DM patients (Table 2). This result is supported by another study by Yuan HL et al. which supported, reporting that an increase in HbA1c is a risk factor for the development of chronic constipation in T_2_DM patients [35]. It can be explained that diabetics with poor blood glucose control suffer from morphological changes in gastrointestinal structure, dysfunction of intestinal motility, dysfunction of intestinal neurons, defect in signal transduction pathways, and accumulation of oxidative stress all of which contribute to constipation [36].

### Strengths and limitations of the study

The study offers crucial insights into the prevalence and associated determinants influencing constipation, as well as it can serve for designing strategies for managing symptoms and averting complications in individuals afflicted with the condition. The ability to examine a lot of variables is also its strength. The study’s constraints include a limited sample and a research design centered on a single facility. It was also challenging to discuss with other related studies in Ethiopia due to shortage of the studies.

## Conclusions

The prevalence of chronic constipation was considerable among T_2_DM patients. Educational status of above high school, sleeping less than seven hours per day, depression, parent history of constipation, water intake less than 1300 ml, TAG and HbAlc play an important role in the development of chronic constipation among T_2_DM patients. Patients with type 2 diabetes can reduce the severity of constipation with timely treatment.

## Data Availability

The datasets used and/or analyzed during the current study are available from the corresponding author on reasonable request.

## Abbreviations

AOR: Adjusted Odd
CI: Confidence Level
COR: Crude Odd Ratio
HbAlc: glycated haemoglobin
HDL: High-Density Lipoprotein: Low-Density Lipoprotein
NGO: Non-Governmental Organization
SD: Standard Deviation
STATA: Statistical Analysis
T2DM: Type 2 Diabetes Mellitus
TAG: Triacylglycerol
TC: Total Cholesterol
